# A targeted genetic association study of epithelial ovarian cancer susceptibility

**DOI:** 10.18632/oncotarget.7121

**Published:** 2016-02-01

**Authors:** Madalene Earp, Stacey J. Winham, Nicholas Larson, Jennifer B. Permuth, Hugues Sicotte, Jeremy Chien, Hoda Anton-Culver, Elisa V. Bandera, Andrew Berchuck, Linda S. Cook, Daniel Cramer, Jennifer A. Doherty, Marc T. Goodman, Douglas A. Levine, Alvaro N.A. Monteiro, Roberta B. Ness, Celeste L. Pearce, Mary Anne Rossing, Shelley S. Tworoger, Nicolas Wentzensen, Maria Bisogna, Louise Brinton, Angela Brooks-Wilson, Michael E. Carney, Julie M. Cunningham, Robert P. Edwards, Zachary C. Fogarty, Edwin S. Iversen, Peter Kraft, Melissa C. Larson, Nhu D. Le, Hui-Yi Lin, Jolanta Lissowska, Francesmary Modugno, Kirsten B. Moysich, Sara H. Olson, Malcolm C. Pike, Elizabeth M. Poole, David N. Rider, Kathryn L. Terry, Pamela J. Thompson, David van den Berg, Robert A. Vierkant, Allison F. Vitonis, Lynne R. Wilkens, Anna H. Wu, Hannah P. Yang, Argyrios Ziogas, Catherine M. Phelan, Joellen M. Schildkraut, Yian Ann Chen, Thomas A. Sellers, Brooke L. Fridley, Ellen L. Goode

**Affiliations:** ^1^ Department of Health Sciences Research, Division of Epidemiology, Mayo Clinic, Rochester, MN, USA; ^2^ Department of Health Sciences Research, Division of Biomedical Statistics and Informatics, Mayo Clinic, Rochester, MN, USA; ^3^ Department of Cancer Epidemiology, H. Lee Moffitt Cancer Center and Research Institute, Tampa, FL, USA; ^4^ Department of Cancer Biology, University of Kansas Cancer Center, Kansas City, KS, USA; ^5^ Department of Epidemiology, University of California Irvine, Irvine, CA, USA; ^6^ Rutgers Cancer Institute of New Jersey and Robert Wood Johnson Medical School, New Brunswick, NJ, USA; ^7^ Duke Cancer Institute, Duke University Medical Center, Durham, NC, USA; ^8^ Division of Epidemiology and Biostatistics, University of New Mexico, Albuquerque, NM, USA; ^9^ Obstetrics and Gynecology Epidemiology Center, Brigham and Women's Hospital and Harvard Medical School, Boston, MA, USA; ^10^ Department of Epidemiology, Harvard School of Public Health, Boston, MA, USA; ^11^ Section of Biostatistics and Epidemiology, The Geisel School of Medicine at Dartmouth, Lebanon, NH, USA; ^12^ Samuel Oschin Comprehensive Cancer Institute, Cedars Sinai Medical Center, Los Angeles, CA, USA; ^13^ Gynecology Service, Department of Surgery, Memorial Sloan-Kettering Cancer Center, New York, NY, USA; ^14^ The University of Texas School of Public Health, Houston, TX, USA; ^15^ Department of Preventive Medicine, Keck School of Medicine, University of Southern California, Los Angeles, CA, USA; ^16^ Department of Epidemiology, University of Washington, Seattle, WA, USA; ^17^ Program in Epidemiology, Division of Public Health Sciences, Fred Hutchinson Cancer Research Center, Seattle, WA, USA; ^18^ Channing Division of Network Medicine, Harvard Medical School and Brigham and Women's Hospital, Boston, MA, USA; ^19^ Division of Cancer Epidemiology and Genetics, National Cancer Institute, Bethesda, MD, USA; ^20^ Genome Sciences Centre, BC Cancer Agency, Vancouver, BC, Canada; ^21^ Department of Biomedical Physiology and Kinesiology, Simon Fraser University, Burnaby, BC, Canada; ^22^ Clinical and Translational Research Program, University of Hawaii Cancer Center, Honolulu, HI, USA; ^23^ Department of Laboratory Medicine and Pathology, Division of Experimental Pathology, Mayo Clinic, Rochester, MN, USA; ^24^ Department of Obstetrics, Gynecology and Reproductive Sciences, Division of Gynecologic Oncology, University of Pittsburgh School of Medicine, Pittsburgh, PA, USA; ^25^ Department of Statistical Science, Duke University, Durham, NC, USA; ^26^ Departments of Epidemiology and Biostatistics, Harvard School of Public Health, Boston, MA, USA; ^27^ Cancer Control Research, BC Cancer Agency, Vancouver, BC, Canada; ^28^ Department of Cancer Epidemiology and Prevention, M. Sklodowska-Curie Memorial Cancer Center & Institute of Oncology, Warsaw, Poland; ^29^ Department of Epidemiology, University of Pittsburgh Graduate School of Public Health, Pittsburgh, PA, USA; ^30^ Cancer Research Program, Magee-Women's Research Institute and University of Pittsburgh Cancer Institute, Pittsburgh, PA, USA; ^31^ Department of Cancer Prevention and Control, Roswell Park Cancer Institute, Buffalo, NY, US; ^32^ Department of Epidemiology and Biostatistics, Memorial Sloan-Kettering Cancer Center, New York, NY, USA; ^33^ Cancer Epidemiology Program, University of Hawaii Cancer Center, Honolulu, HI, USA; ^34^ Department of Epidemiology, Center for Cancer Genetics Research and Prevention, School of Medicine, University of California Irvine, Irvine, CA, USA; ^35^ Department of Community and Family Medicine, Duke University Medical Center, Durham, NC, USA; ^36^ Cancer Prevention, Detection and Control Research Program, Duke Cancer Institute, Durham, NC, USA; ^37^ Kansas IDeA Network of Biomedical Research Excellence Bioinformatics Core, University of Kansas Cancer Center, Kansas City, KS, USA

**Keywords:** ovarian cancer, high-grade serous carcinoma, genetic association, susceptibility loci, NF-κB

## Abstract

**Background:**

Genome-wide association studies have identified several common susceptibility alleles for epithelial ovarian cancer (EOC). To further understand EOC susceptibility, we examined previously ungenotyped candidate variants, including uncommon variants and those residing within known susceptibility loci.

**Results:**

At nine of eleven previously published EOC susceptibility regions (2q31, 3q25, 5p15, 8q21, 8q24, 10p12, 17q12, 17q21.31, and 19p13), novel variants were identified that were more strongly associated with risk than previously reported variants. Beyond known susceptibility regions, no variants were found to be associated with EOC risk at genome-wide statistical significance (*p* <5×10^−8^), nor were any significant after Bonferroni correction for 17,000 variants (*p*< 3×10-6).

**Methods:**

A customized genotyping array was used to assess over 17,000 variants in coding, non-coding, regulatory, and known susceptibility regions in 4,973 EOC cases and 5,640 controls from 13 independent studies. Susceptibility for EOC overall and for select histotypes was evaluated using logistic regression adjusted for age, study site, and population substructure.

**Conclusion:**

Given the novel variants identified within the 2q31, 3q25, 5p15, 8q21, 8q24, 10p12, 17q12, 17q21.31, and 19p13 regions, larger follow-up genotyping studies, using imputation where necessary, are needed for fine-mapping and confirmation of low frequency variants that fall below statistical significance.

## INTRODUCTION

Epithelial ovarian cancer (EOC) is the second most common gynecologic cancer in the US, but it leads in deaths owing to its tendency to be diagnosed in the late stages of disease [1]. EOC is composed of five major histologic types [2]: high-grade serous carcinoma (HGSC), accounting for most cases (∼70%); and the rarer clear cell, endometrioid, mucinous, and low-grade serous carcinomas (LGSC). Known rare mutations in DNA repair and mismatch repair genes are thought to account for 10%-15% of all EOCs [3-9]. Common alleles identified by genome-wide association studies (GWAS) are thought to account for an additional 3%-4% of EOC risk [10-17]. Still, much of about the heritability of EOC remains unaccounted for. Here, we sought to identify additional EOC susceptibility variants through direct genotyping and analysis of EOC cases and controls from 13 independent studies. We targeted variants based on innovative pilot studies, hypothesizing that previously ungenotyped variants may be responsible for a proportion of the unexplained EOC susceptibility.

## RESULTS

### Known EOC susceptibility regions

**Figure 1:**
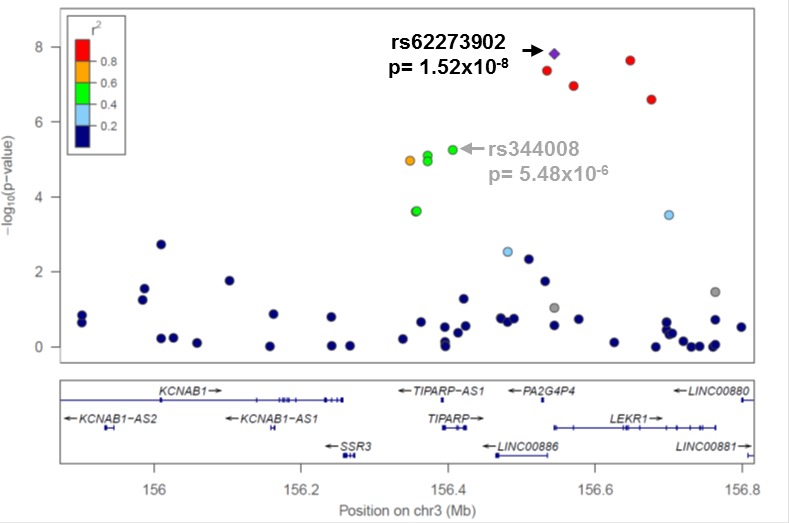
Novel variant rs62273902 in the 5′-untranslated region of LEKR1 has the strongest association signal at 3q25 Regional association plot for variants genotyped at 3q25 in all EOC histologies cases (*N* = 4,973) and controls (*N* = 5,640). Linkage disequilibrium between rs62273902 and each variant is estimated using data from 5,640 controls and indicated by the color scheme. The previously reported risk variant rs2665390 in this region {Goode, 2010 #23} was not genotyped; rs344008 (*p* = 5×10^−6^) is indicated in its place to allow comparison of the novel (rs62273902) and known (rs2665390) most associated variants (r^2^ = 1 for rs344008 and rs2665390 in 1000 Genomes Project phase 1 European data).

**Figure 2:**
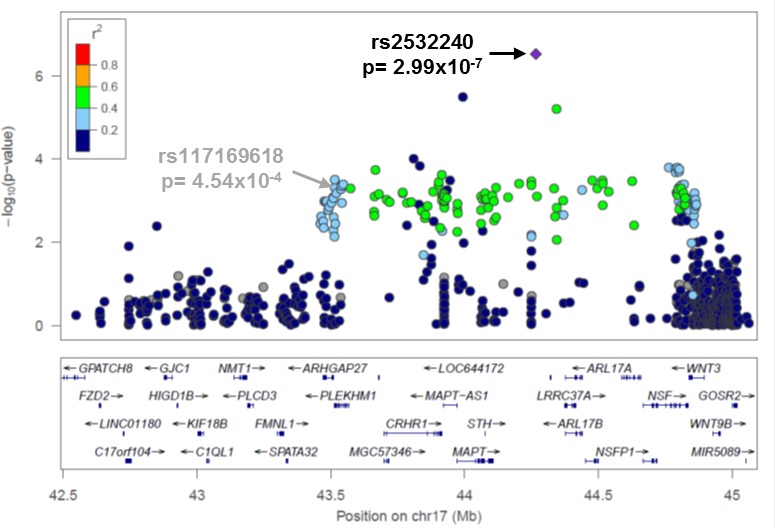
Novel variant rs2532240 has the strongest association signal at 17q21.31 Regional association plot for variants genotyped at 17q21.31 in all EOC histologies cases (*N* = 4,973) and controls (*N* = 5,640). The most associated variant was rs2532240 (*p* = 3 × 10^−7^). Linkage disequilibrium between rs2532240 and each variant is estimated using data from 5,640 controls and indicated by the color scheme. The previously reported risk variant rs12942666 in this region {Permuth-Wey, 2013 #28} was not genotyped, but rs117169618 (*p* = 5 × 10^−4^) is indicated in its place to allow comparison of the novel (rs2532240) and known (rs12942666) most variants (r^2^ = 0.8 for rs117169618 and rs12942666 in 1000 Genomes Project phase 1 European data).

One goal of this project was to compare the relative strength of the associations between known and novel variants within the first eleven published EOC risk loci [10-12, 14, 18] (Supplemental Table 1). The variants most strongly associated with EOC risk in this study (all histology or HGSC) are given in Table 1 and plotted regionally in Figure 1, Figure 2, and Supplemental Figure 1. Compared to published variants, novel variants were more strongly associated with susceptibility of all histologies of EOC at nine loci (2q31, 3q25, 5p15, 8q21, 8q24, 10p12, 17q12, 17q21.31, and 19p13) (Table 1); all but three (8q21, 17q21.31, and 19p13) are in moderate LD (r^2^> 0.4) with known variants (Supplemental Figure 1). At the 3q25 locus variant rs62273902 (p_all-histology_= 2 × 10^−8^) was the most strongly associated variant (Figure 1), and at the 17q21.31 locus variant rs2532240 (p_all-histology_= 3 × 10^−7^) was the most strongly associated variant (Figure 2). In the HGSC only analysis, novel variants were more strongly associated with susceptibility at seven loci (2q31, 3q25, 8q24, 10p12, 17q12, 17q21.31, and 19p13; Table 1); all but two (2q31, 17q21.31) are in moderate LD (r^2^> 0.4) with known variants (Supplemental Figure 1). With two exceptions (noted below), novel variants were common (minor allele frequency (MAF)>0.05), in the intron of genes or intergenic, in moderate-to-strong LD with known variants, and conferred modest effects on susceptibility. One exception was the association of rare intergenic variant rs74955251 at 8q21 (MAF_overall_= 0.00028, OR= 3.9 × 10^−6^, 95% confidence interval [CI]: 3.4 × 10^−118^-4.6 × 10^106^, p_all-histology_= 4 × 10^−3^). Given its rarity, rs74955251 requires assessment in a much larger sample of cases and controls. A second exception was the association of common (MAF_overall_= 0.50) missense variant rs2363956 in the gene *ANKLE1* at 19p13 (OR= 0.91, 95% CI: 0.87-0.97, p_all-histology_ = 2 × 10^−3^, protein change Leu184Trp).

**Table 1:** Most significant associations within eleven known EOC susceptibility regions

	All histologies (4,973 cases, 5,640 controls)						High grade serous (3,573 cases, 5,640 controls)					
Region	Variant	Position (hg19)	Nearest Gene	MAF overall	OR (95% CI)	P value	Variant	Position (hg19)	Nearest Gene	MAF overall	OR (95% CI)	P value
2q31	rs711830	177037311	3′ UTR of *HOXD3*	0.33	1.12 (1.05-1.18)	2.05 × 10^−4^	rs1374325	177043971	Intron of *HOXD1-AS*	0.32	0.89 (0.83-0.96)	1.37 × 10^−3^
3q25	rs62273902	156544488	5′ UTR of *LEKR1*	0.06	1.40 (1.25-1.57)	1.52 × 10^−8^	rs62275810	156647851	Intron of *LEKR1*	0.05	1.46 (1.27-1.68)	2.58 × 10^−7^
5p15	rs4975538	1280830	Intron of *TERT*	0.35	1.13 (1.06-1.20)	8.24 × 10^−5^	**rs10069690**	1279790	Intron of *TERT*	0.26	1.14 (1.06-1.22)	6.28 × 10^−4^
8q21	rs74955251	82230643	34kb 3′ of *FABP5*	2.8 × 10^−4^	3.94 × 10^−6^ (3.4 × 10^−118^-4.6 × 10^106^)	5.71 × 10^−3^	**rs11782652**	82653644	Intron of *CHMP4C*	0.07	1.23 (1.09-1.39)	6.49 × 10^−4^
8q24	rs1400482	129541931	Intron of *LINC00824*	0.12	0.82 (0.75-0.89)	2.57 × 10^−6^	rs1400482	129541931	Intron of *LINC00824*	0.13	0.80 (0.73-0.89)	1.71 × 10^−5^
9p22	**rs3814113**	16915021	44kb 5′ of *BNC2*	0.32	0.81 (0.76-0.86)	7.96 × 10^−13^	**rs3814113**	16915021	44kb 5′ of *BNC2*	0.32	0.75 (0.70-0.81)	3.20 × 10^−5^
10p12	rs4364959	22285371	Intron of *DNAJC1*	0.30	1.15 (1.08-1.22)	9.66 × 10^−6^	rs9971210	21879084	Intron of *MLLT10*	0.49	1.11 (1.04-1.19)	1.21 × 10^−3^
17q12	rs7405776	36093022	Intron of *HNF1B*	0.38	1.18 (1.11-1.24)	1.12 × 10^−8^	rs7405776	36093022	Inton of *HNF1B*	0.37	1.25 (1.17-1.34)	2.23 × 10^−11^
17q21.31	rs2532240	44265839	Intron of *KANSL1*	0.41	1.16 (1.10-1.23)	2.99 × 10^−7^	rs3785880	43993376	Intron of *MAPT*	0.45	0.87 (0.82-0.93)	4.95 × 10^−5^
17q21.32	**rs9303542**	46411500	Intron of *SKAP1*	0.29	1.15 (1.08-1.23)	8.39 × 10^−6^	**rs9303542**	46411500	Intron of *SKAP1*	0.27	1.16 (1.08-1.25)	4.88 × 10^−5^
19p13	rs2363956	17394124	Missense mutation (L184W) in *ANKLE1*	0.50	0.91 (0.87-0.97)	1.57 × 10^−3^	rs56069439	17393925	Intron of *ANKLE1*	0.30	1.15 (1.07-1.23)	1.82 × 10^−4^

Bolded variants were previously reported as the most strongly associated variants in these susceptibility regions (Supplemental Table 1). MAF, minor allele frequency; OR, odds ratio; CI, confidence interval; UTR, untranslated region. Associations adjust for age, site, and three European principal components.

### Beyond known EOC susceptibility regions

We targeted 5,320 variants which showed suggestive association with susceptibility in a pilot-scale whole genome sequence analysis that compared germline sequence of EOC patients (N= 19) to 1000 GP participants (N= 174). No novel variants reached genome-wide significance for association with EOC risk overall or HGSC (p≤ 5×10^−8^), nor were significant after Bonferroni correction (p≤ 9×10^−6^). Nonetheless, the risk estimates generally were in the expected direction based on pilot data, and several variants merit investigation in larger case-control collections (Table 2). For example, among variants targeted because they were present only in EOC germline sequence data (WGS EOC+ in Table 2), the most strongly associated risk variant was rs138643956 (OR= 3.68; p_HGSC_= 2 × 10^−4^). For variants selected because they were absent from whole genome sequenced EOC cases (WGS EOC- in Table 2), the most associated variant was rs9380516 (OR= 0.83; p_HGSC_= 6 × 10^−5^); in the current study this variant showed a case MAF of 0.15, suggesting that this was a missed variant in the pilot sequencing study. For variants targeted which were present in whole genome sequenced EOC cases and in 1000 GP data, but differed in MAF (WGS EOC↑ and WGS EOC↓ in Table 2), the current analyses were generally consistent, including rs117841616 on chromosome 20 (p_all histology_= 2 × 10^−4^) and rs240783 (p_HGSC_= 8 × 10^−4^) on chromosome 6. In general, very few variants targeted based on suggestive association in pilot sequence study had appreciable MAF differences (case vs. control) in the current genotyping study. As expected due to small sample size, we observed that case MAF estimates in our pilot whole genome sequencing study were both inflated and deflated compared to the current study.

**Table 2:** Most significant EOC risk associations by selection criteria outside of eleven known susceptibility regions

					Pilot WGS Study		Case-control study data (13 sites)					
Selection Criteria	Variant	Chr.	Position (hg19)	Location	EOC MAF	1000 GP MAF	Histology	Case MAF	Control MAF	N case, N control	OR (95%CI)	P value
WGS EOC+	rs138643956	10	79367857	Intron of *KCNMA1*	0.053	0.000	HGSC	0.004	0.001	3035, 5637	3.68 (1.79 - 7.55)	1.85 × 10^−4^
WGS EOC↑	rs117841616	20	57855211	20kb 5′ of *EDN3*	0.079	0.006	All	0.008	0.005	4973, 5634	1.93 (1.36 - 2.74)	2.06 × 10^−4^
WGS EOC↓	rs240783	6	100968737	Intron of *ASCC3*	0.184	0.494	HGSC	0.400	0.428	2956, 5518	0.89 (0.84 - 0.95)	7.90 × 10^−4^
WGS EOC−	rs9380516	6	35502202	10kb 5′ of *RP3-340B19.3*	0.000	0.155	HGSC	0.147	0.171	3027, 5633	0.83 (0.76 - 0.91)	6.44 × 10^−5^
NF-κB	rs10143322	14	91556577	24kb 5′ of *C14orf159*	n.a.	n.a.	All	0.220	0.246	4562, 5634	0.87 (0.82 - 0.93)	2.99 × 10^−5^
NF-κB	rs6092485	20	56045014	26kb 3′ of *CTCFL*	n.a.	n.a.	HGSC	0.335	0.311	3019, 5625	1.13 (1.05 - 1.21)	6.77 × 10^−4^
Endometrioid GWAS	rs9264042	6	31196801	*25kb 3′ of HCG27*	n.a.	n.a.	All	0.125	0.107	4440, 5505	1.17 (1.07 - 1.28)	4.71 × 10^−4^
Endometrioid GWAS	rs2638653	8	18666210	Intron of *PSD3*	n.a.	n.a.	EC	0.409	0.362	832, 5619	1.23 (1.11 - 1.37)	1.28 × 10^−4^

WGS, whole-genome sequencing; EOC, epithelial ovarian cancer; 1000 GP, 1000 Genomes Project; GWAS, genome wide association study; Chr, chromosome; MAF, minor allele frequency; OR, odds ratio; CI, confidence interval; HGSC, high grade serous carcinoma; n.a., not applicable. WGS EOC+ variant selection criteria: MAF> 0% in serous EOC cases, monomorphic in 1000 GP European individuals; WGS EOC↑ variant selection criteria: polymorphic in cases and 1000 GP, MAF greater in WGS patients; WGS EOC↓ variant selection criteria: polymorphic in cases and 1000 GP, MAF greater in 1000 GP; WGS 1000 EOC− variant selection criteria: monomorphic in cases, MAF> 0% in 1000 GP.

Finally, among NF-κB-related variants and those hypothesized to associate with endometrioid EOC risk, the most suggestive results for variants which disrupt NF-κB binding [19, 20] were intergenic variants rs10143322 on chromosome 14 (p_all-histology_= 3 × 10^−5^) and rs6092485 on chromosome 20 (p_HGSC_= 7×10^−4^) (Table 2). If Bonferroni correction for the number NF-κB binding site variants tested is applied, the threshold for statistical significance is p<4 × 10^−5^ (p= 0.05/1,302), and this single variant, rs10143322, is declared significant; using experiment-wide and certainly genome-wide multiple testing corrections, it is not significant. Among variants previously identified in a pilot GWAS of endometrioid EOC, the most significant variants were intronic variant rs2638653 (p_endometrioid_= 1 × 10^−4^) on chromosome 8 (Table 2), and intergenic rs9264042 on chromosome 6 (p_all-histology_= 5 × 10^−4^). These modest associations may also warrant follow-up in larger studies.

## DISCUSSION

The objective of this study was to test whether novel variants identified through a combination of approaches were associated with EOC susceptibility. We took an innovative approach to the selection of variants, including the use of whole genome sequencing data to target novel variants correlated with known GWAS risk variants, comparison of sequencing EOC cases to 1000 GP participants beyond these regions, NF-κB functional data, and GWAS analysis of EOC cases with endometrioid histology.

In nine of the eleven susceptibility regions investigated, novel variants were more highly associated with all histology EOC risk than previously reported variants, and, in the HGSC only analysis, novel variants were more strongly associated with susceptibility at seven loci. Further work on thesevariant may provide more biological insight. For example, at the 3q25 locus, the novel variant rs62273902 (all histology) coincides with a genomic sequence that appears functionally active in a range of cell lines and tissues relevant to EOC, including ovary, as assayed by the Roadmap Epigenomics Mapping Consortium (REMC), http://www.epigenomebrowser.org/. rs62273902 resides within a DNase peak, an active transcription start site (TSS), and multiple proteins across diverse tissues bind the sequence spanning this variant. rs62273902 is therefore a good functional candidate variant at this locus. As well, at 17q21.31, the novel variants (rs2532240 in all histologies and rs3785880 in HGSC-only analysis) are separated by 272 kb and not correlated with each other or the previously reported variant rs1294266. rs2532240 coincides with a chromatin region marked as a weak/poised enhancer in several tissues, including ovary (REMC [21]); however, it does not overlap transcription factor binding sites (TFBS) or DNase peaks. rs12942666 does not coincide with promoter or enhancer regions in tissues relevant to EOC in the REMC data, suggesting it is unlikely to be functionally relevant. The 17q21.31 variants are located in a large region of strong LD previously identified as the “17q21.31 inversion” (∼900kb long), which exists either as a direct (H1) or inverted (H2) haplotype in the European population [14, 15, 22]. Further investigation of how these variants might impact EOC risk is needed.

Of critical note, a large EOC meta-GWAS with imputation to revised Phase I 1000 GP data was recently completed with over 23,000 cases and 35,000 controls of the Ovarian Cancer Association Consortium, including many of the participants in the current analysis. We inspected our most associated variants from the 11 known susceptibility regions in in an online look-up of results based on these data (http://apps.ccge.medschl.cam.ac.uk/consortia/ocac/contact/contact.html). In general, the variants reported here were highly ranked in the EOC meta-GWAS data (i.e., in the top 50 most associated variants in the regions we defined). At 8q24, the novel directly genotyped variant presented here (rs1400482) was the most associated variant in the larger imputation-based study. At 3q25, 10p12, 17q21.31, and 17q21.32, the most significant variant in the current study was not among the most significant in the imputation-based study. Nonetheless, novel variants at 3q25 and 17q21.32 remained highly significant (rs62273902 at 3q25 p_meta_= 2 × 10^−28^, and rs9303542 at 17q21.32 p_meta_= 3 × 10^−12^). Although novel variants at 3q25 and 17q21.32 remained highly significant (rs62273902 at 3q25 p_meta_= 2 × 10^−28^, and rs9303542 at 17q21.32 p_meta_= 3 × 10^−12^), at these two regions as well as at 10p12 and 17q21.31, the imputation-based study revealed stronger associations with other variants.

Among variants genotyped based on our pilot study comparing whole genomes of EOC cases and 1000 GP participants, none were significant after multiple testing correction for 5,320 variants. Despite our sample size (4,973 cases and 5,640 controls), power to detect associations with low MAF variants was limited. Variants in NF-κB binding sites were also not associated with EOC risk at genome-wide significance. Noting the debate regarding the use of p<5 × 10^−8^ as the threshold for statistical significance when evaluating potentially functional variants with presumed higher prior probability [23], Bonferroni correction for the number of NF-κB binding site variants yields one statistically significant variant (rs10143322, p= 3×10^−5^). rs2638653, a variant selected based on an unpublished GWAS of endometrioid EOC, and found here to be the variant most associated with endometrioid EOC risk (p= 1 × 10^−4^), coincides with chromatin marked as being an active promoter in ovary tissues (of *PSD3*), but not an enhancer or DNase site. Interestingly, loss of heterozygosity on chromosome 8p22, where this variant is located, is common in EOC tumors, and reduced expression of genes in this region has been found to negatively impact survival in EOC [24].

In summary, we developed a diverse panel of previously ungenotyped variants to directly test for association with EOC susceptibility in 4,973 EOC cases and 5,640 controls from 13 independent studies. Our innovative approach to variant selection included the first use of whole-genome sequencing data from EOC cases in novel variant discovery. At several EOC susceptibility regions, we report novel risk variants for further association and functional investigation. Beyond known regions, this first pass at using whole genome sequencing pilot analyses, although underpowered, also yielded variants of potential interest (rs138643956 and rs117841616). The key strength of this report is the use of direct genotyping of novel variants, some rare, while its key limitation is an inability to more comprehensively examine rare variation. Larger scale genotyping and/or improved genotype imputation accuracy will facilitate further scrutiny of the variants highlighted here.

## MATERIALS AND METHODS

### Study participants

Study participants were drawn from 13 independent EOC case-control studies of the Ovarian Cancer Association Consortium and were restricted to women of European ancestry. Characteristics of the contributing studies are given in Supplemental Table 2 and have been described previously [18]. Cases (N=4,973) consisted of women aged 18 and older with a pathologically confirmed primary invasive EOC, fallopian tube cancer, or primary peritoneal cancer; controls (N=5,640) were matched by age and region.

### Genotyping array

A total of 17,439 germline DNA variants were genotyped using a customized Affymetrix Axiom Exome array (Affymetrix Corporation, Santa Clara, CA). These variants were drawn from four discovery categories: 1) from eleven known EOC susceptibility regions (N =6,948; Supplemental Table 3) [10-14, 18], identified by *in silico* fine-mapping and a small germline whole genome sequencing study of EOC cases, 2) variants outside these eleven regions which showed suggestive association in pilot whole genome sequencing of serous EOC cases (compared to 1000 Genomes Project [GP] data)(N = 7,189), 3) variants with a hypothesized role in disrupted binding of NF-κB transcription factors, which are known to have central roles in immune and inflammatory responses and cancer [19, 20, 25, 26](N = 1,302), and 4) the top associated variants from a pilot GWAS of endometrioid EOC (N = 2,000). See the Supplemental Methods for more detail on the selection of these variants.

### Quality control

Germline DNA was genotyped at the Affymetrix Research Services Laboratory (Santa Clara, CA) using default quality control (QC) and genotype calling criteria. Variants failed QC if: (1) the call rate was < 95%; (2) p-values of Hardy-Weinberg equilibrium in controls were < 10^−7^; or (3) there was > 2% discordance in duplicate pairs. Further, monomorphic variants were removed. Of 6,948 variants genotyped within 11 known EOC risk regions, 4,919 (71%) met these QC criteria and were polymorphic. Outside of these regions, of 7,189 variants selected based on whole-genome sequencing data, 5,286 (74%) met QC criteria and were polymorphic. Of 1,302 variants associated with NF-κB binding, 980 (75%) met QC criteria and were polymorphic, and, of 2,000 variants selected from a GWAS of endometrioid EOC, 1,826 (91%) met QC criteria and were polymorphic. Most variants were excluded for being monomorphic. Thus, a total of 13,011 genotyped variants remained for analysis.

### Association analysis

All cases were included in the overall EOC risk association analyses (N=4,973). Subset analyses were performed on histologic subsets based on *a priori* selection; HGSC (N=3,573) and endometrioid EOC (N=835). For each analysis, 5,640 controls were used. Associations were estimated using logistic regression assuming an additive genetic model, adjusting for age, study site, and population substructure by including the first three eigenvalues from principal components analysis [18]. All analyses were conducted in R version 3.0.2 (http://www.R-project.org/).

## SUPPLEMENTARY MATERIAL METHODS FIGURE AND TABLES


