# Supplementary material for: A targeted genetic association study of epithelial ovarian cancer susceptibility
Source: Oncotarget. 2016 Feb 1;7(7):7381–9. doi: 10.18632/oncotarget.7121 (PMC4884925; doi:10.18632/oncotarget.7121)
Supplement: Supplementary file 1 [file oncotarget-07-7381-s001.pdf]

A targeted genetic association study of epithelial ovarian cancer susceptibility

Supplementary Material

Supplemental Methods

Selection of variants within eleven known EOC susceptibility regions

Data from 25 individuals diagnosed with ovarian or peritoneal (N=1) cancer (17 high grade serous carcinoma [HGSC], 3 clear cell, 3 endometrioid, 1 mucinous, 1 low grade serous carcinoma [LGSC]) was used to select custom variants at 11 EOC susceptibility regions (see **Supplemental Table 1 & 3**). Germline whole genome sequencing was performed using an Illumina GAIIx, providing a mean of 48-fold coverage and with 86% of the genome on average having at least 10-fold coverage [1]. Variants considered for inclusion on the array were those in linkage disequilibrium (LD, $r^2 \geq 0.2$) with the most strongly associated published risk variants, and in the 20kb flanking sequence of these variants. Variants were excluded if present in HapMap Phase II[2] or on the Illumina Collaborative Oncology Gene-environment Study[3] array because the intended focus was previously ungenotyped rarer variants. A total of 7,901 variants met these criteria and fell into 1,759 clusters of variants in perfect LD. For each cluster, one variant was selected for genotyping if cluster size was <5 variants, two variants were selected if cluster size was 5-10 variants, and three variants if cluster size was >10 variants. A total of 778 variants were selected in this way. Additional custom variants within these 11 regions were also added to the array, including the known EOC risk variants, and variants from an unpublished HGSC GWAS. Variants residing within these 11 genomic regions that were part of the standard array content on the Affymetrix Axiom Exome array (Affymetrix Corporation, Santa Clara, CA) are also included in this report.

Whole genome sequencing variants outside of known EOC susceptibility regions

Serous EOC cases that were whole genome sequenced (18 HGSC and 1 LGSC; **Supplemental Table 3**) were also used to select novel variants outside of the susceptibility regions as described above. Our aim was to identify rare variants. In brief, whole genome sequencing data identified 800,000 variants, whose

minor allele frequency (MAF) was compared to 1000 Genomes Project (GP) data for 174 Europeans (CEU/GBR, version 20110521) who were assumed unaffected by EOC. A Z-test for equal proportions was used to assess variants for association with EOC risk, and rank them according to p-value. Rather than select these variants based solely on extreme p-values, which may reflect sequence platform differences or the small sample size of the study, variants were placed in one of four categories based on MAF and each category was over-sampled. The four categories were: 1) MAF > 0 in serous EOC cases, monomorphic in 1000 GP ("EOC+"); 2) polymorphic in cases and 1000 GP, but MAF greater in cases ("EOC↑"); 3) polymorphic in cases and 1000 GP, but MAF less in cases ("EOC↓"); and 4) monomorphic in cases, MAF> 0 in 1000 GP ("EOC−"). In total, we selected the 1,800 custom variants from each category with the lowest p-values (7,200 in total). After satisfying the Affymetrix probe design criteria, 7,189 variants were included on the array.

NF-κB and endometrioid EOC GWAS variants

We included 1,170 variants in NF-κB binding motifs that were validated or predicted to disrupt binding[4, 5] and 132 variants surrounding SNPs in NF-κB related candidate genes that were previously associated with EOC risk[6] as custom content. We also included 2,000 of the most significantly associated variants from an unpublished GWAS among 1,452 endometrioid EOC cases.

References for Supplemental Methods

1. Chien J SH, Fan JB, Humphray S, Cunningham JM, Kalli KR, Oberg AL, Hart S, Li Y, Davila JI, Baheti S, Wang C, Kocher J-PA, Dietmann S, Atkinson EJ, Asmann Y, Bell D, Ota T, Tarabishy Y, Kuang R, Bibikova M, Cheetham RK, Grocock RJ, Swisher EM, Peden J, Bentley D, Kaufmann S, Hartmann LC, Shridhar V, and Goode EL. TP53 mutations, tetraploidy, and homologous cecombination repair defects in early stage high grade serous ovarian cancer. Nucleic Acids Res. 2015.

2. Frazer KA, Ballinger DG, Cox DR, Hinds DA, Stuve LL, Gibbs RA, Belmont JW, Boudreau A, Hardenbol P, Leal SM, Pasternak S, Wheeler DA, Willis TD, Yu F, Yang H, Zeng C, et al. A second generation human haplotype map of over 3.1 million SNPs. Nature. 2007; 449(7164):851-861.

3. Pharoah PD, Tsai YY, Ramus SJ, Phelan CM, Goode EL, Lawrenson K, Buckley M, Fridley BL, Tyrer JP, Shen H, Weber R, Karevan R, Larson MC, Song H, Tessier DC, Bacot F, et al. GWAS meta-analysis and replication identifies three new susceptibility loci for ovarian cancer. Nat Genet. 2013; 45(4):362-370.

4. Kasowski M, Grubert F, Heffelfinger C, Hariharan M, Asabere A, Waszak SM, Habegger L, Rozowsky J, Shi M, Urban AE, Hong MY, Karczewski KJ, Huber W, Weissman SM, Gerstein MB, Korbel JO, et al. Variation in transcription factor binding among humans. Science. 2010; 328(5975):232-235.

5. Karczewski KJ, Tatonetti NP, Landt SG, Yang X, Slifer T, Altman RB and Snyder M. Cooperative transcription factor associations discovered using regulatory variation. Proc Natl Acad Sci U S A. 2011; 108(32):13353-13358.

6. Charbonneau B, Block MS, Bamlet WR, Vierkant RA, Kalli KR, Fogarty Z, Rider DN, Sellers TA, Tworoger SS, Poole E, Risch HA, Salvesen HB, Kiemeney LA, Baglietto L, Giles GG, Severi G, et al. Risk of ovarian cancer and the NF-kappaB pathway: genetic association with IL1A and TNFSF10. Cancer Res. 2014; 74(3):852-861.

Supplemental Figure 1

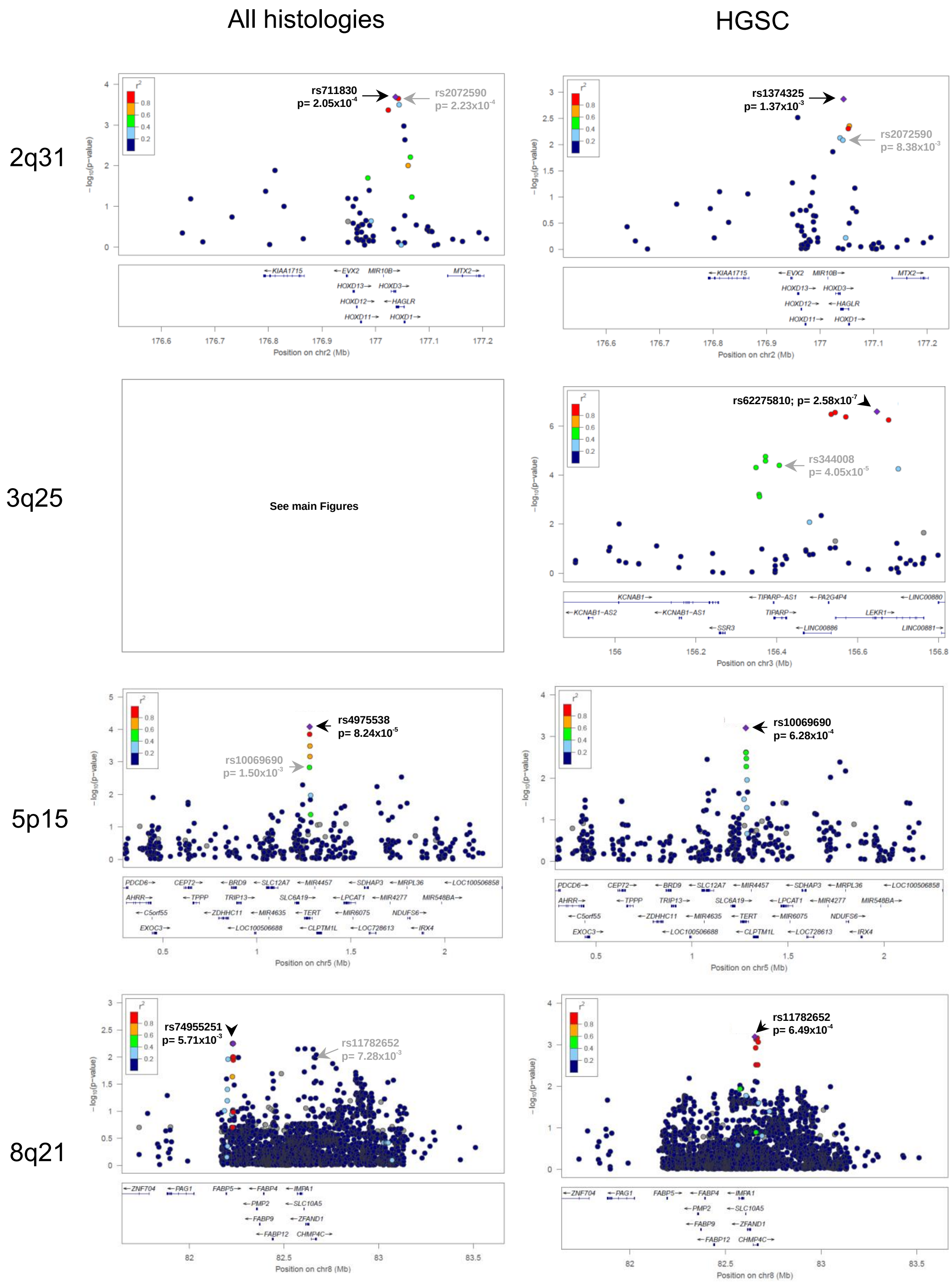

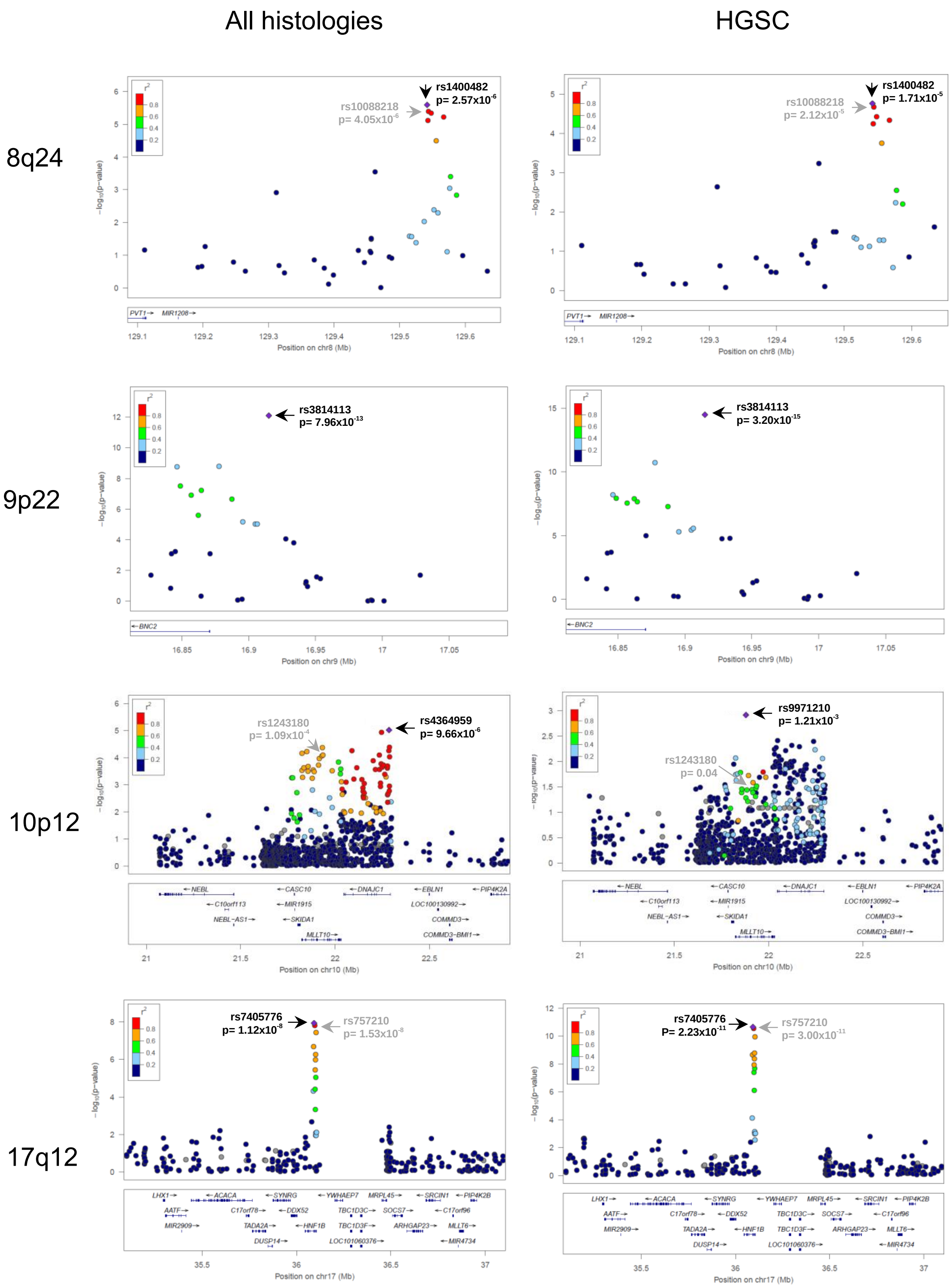
All histologies
HGSC
8q24
rs1400482
p= 2.57x10-6
rs10088218
p= 4.05x10-6
rs1400482
p= 1.71x10-5
rs10088218
p= 2.12x10-5
Position on chr8 (Mb)
9p22
rs3814113
p= 7.96x10-13
rs3814113
p= 3.20x10-15
Position on chr9 (Mb)
10p12
rs1243180
p= 1.09x10-4
rs4364959
p= 9.66x10-6
rs9971210
p= 1.21x10-3
rs1243180
p= 0.04
Position on chr10 (Mb)
17q12
rs7405776
p= 1.12x10-8
rs757210
p= 1.53x10-8
rs7405776
P= 2.23x10-11
rs757210
p= 3.00x10-11
Position on chr17 (Mb)

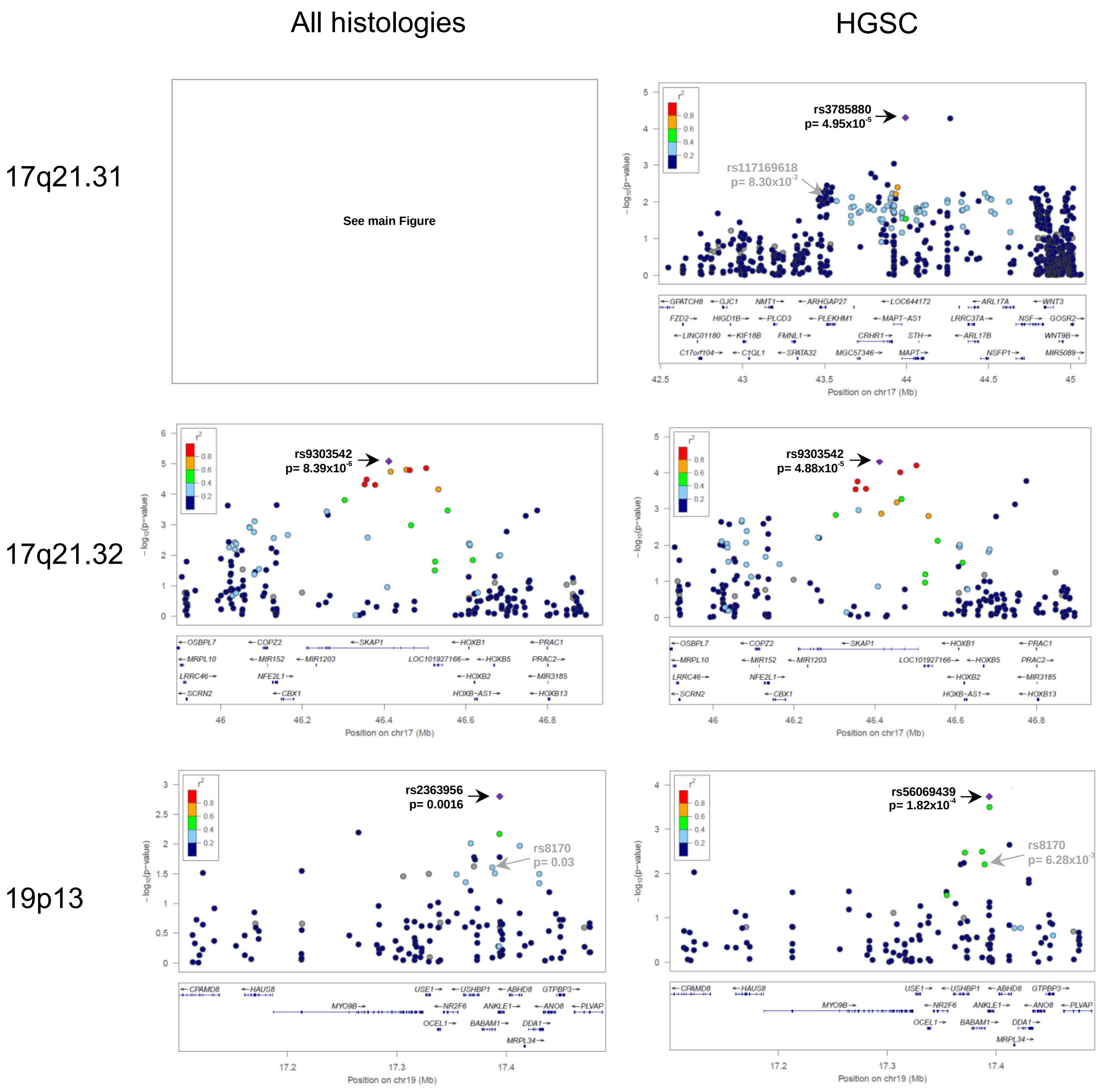

All histologies
HGSC
17q21.31
17q21.32
19p13
See main Figure
rs3785880
p= 4.95x10^{-5}
rs117169618
p= 8.30x10^{-3}
rs9303542
p= 8.39x10^{-6}
rs9303542
p= 4.88x10^{-5}
rs2363956
p= 0.0016
rs8170
p= 0.03
rs56069439
p= 1.82x10^{-4}
rs8170
p= 6.28x10^{-3}
Position on chr17 (Mb)
Position on chr19 (Mb)

Supplemental Table 1. Eleven previously reported EOC susceptibility regions: prior association results that guided current variant selection.

Locus	Prior most associated variant	Position	Reported P-value	Histotype	Reference	Region investigated in the current study (size)	Number of variants genotyped
2q31	rs2072590	177042633	5×10^{-11}	All	Goode et al., 2010	176530334-177230512 (0.7 Mb)	66
3q25	rs2665390	156397749	7×10^{-8}	Serous	Goode et al., 2010	155882491-156806437 (0.9 Mb)	63
5p15	rs10069690	1279790	1×10^{-11}	Serous	Bojensen et al., 2013	296759-2296759 (2.0 Mb)	352
8q21	rs11782652	82653644	7×10^{-10}	Serous	Pharoah et al., 2013	81653644-83653644 (2.0 Mb)	2,141
8q24	rs10088218	129543949	8×10^{-15}	Serous	Goode et al., 2010	129095188-129644067 (0.5 Mb)	43
9p22	rs3814113	16915021	4×10^{-21}	Serous	Song et al., 2009	16821349-17083703 (0.3 Mb)	28
10p12	rs1243180	21915619	2×10^{-8}	All	Pharoah et al., 2013	20915619-22915619 (2.0 Mb)	850
17q12	rs757210	36096515	8×10^{-10}	Serous	Pharoah et al., 2013	35096515-37096515 (2.0 Mb)	261
17q21.31	rs12942666	43499839	3×10^{-10}	Serous	Permuth-Wey et al., 2013	42499839-45083402 (2.6 Mb)	786
17q21.32	rs9303542	46411500	1×10^{-7}	Serous	Goode et al., 2010	45901906-46925056 (1.0 Mb)	197
19p13	rs8170	17389704	4×10^{-11}	Serous	Bolton et al., 2010	17110400-17481000 (0.4 Mb)	132

The genotyping array was designed in mid-2012 based on the most strongly associated variants known at the time. Mb, megabase; Build 37.

Supplemental Table 2. Characteristics of study participants

	Cases (N=4,973)	Controls (N=5,640)
**Study: region**		
NEC: New Hampshire and Massachusetts, USA	1,210 (24%)	1,708 (30%)
DOV: Washington, USA	789 (16%)	796 (14%)
MAY: Upper Midwest, USA	645 (13%)	713 (13%)
NCO: Central/Eastern North Carolina, USA	456 (9%)	459 (8%)
USC: Los Angeles County, USA	415 (8%)	461 (8%)
OVA: Alberta and British Columbia, Canada	372 (8%)	409 (7%)
HOP: Northeast, USA (PA, OH, NY)	370 (7%)	383 (7%)
UCI: Southern California, USA	160 (3%)	160 (3%)
NHS: USA	139 (3%)	162 (3%)
MSK: New York City, NY, USA	132 (3%)	161 (3%)
POL: Warszaw and Lodz, Poland	127 (3%)	59 (1%)
NJO: New Jersey, USA	102 (2%)	105 (2%)
HAW: Hawaii, USA	56 (1%)	64 (1%)
**Age, years**		
Mean (Range)	59 (21-91)	58 (19-91)
**Site (cases only)**		
ovary	4,570 (92%)	n.a.
primary peritoneum	341 (7%)	n.a.
fallopian tube	62 (1%)	n.a.
**Grade (cases only)**		
well differentiated	425 (10%)	n.a.
moderately differentiated	861 (21%)	n.a.
poorly differentiated	2,260 (55%)	n.a.
undifferentiated	536 (13%)	n.a.
unknown	891	n.a.
**Stage (cases only)**		
localized	536 (14%)	n.a.
regional	567 (14%)	n.a.
distant	2,838 (72%)	n.a.
unknown	1032	n.a.
**Histotype (cases only)**		
high grade serous	3,573 (75%)	n.a.
endometrioid	835 (18%)	n.a.
low grade serous	152 (3%)	n.a.
clear cell	121 (3%)	n.a.
mucinous	71 (1%)	n.a.
other, unknown	221	n.a.

n.a., not applicable

Supplemental Table 3. Tumor characteristics of White germline whole-genome sequenced EOC patients from the Mayo Clinic.

Site	Histotype	Grade	FIGO stage	Age at diagnosis
ovary	High-grade serous	4, undifferentiated	IA	66
peritoneum	High-grade serous	4, undifferentiated	IIB	81
ovary	High-grade serous	4, undifferentiated	IIB	50
ovary	High-grade serous	4, undifferentiated	IIC	55
ovary	High-grade serous	4, undifferentiated	IIC	84
ovary	High-grade serous	4, undifferentiated	IIC	77
ovary	High-grade serous	2, moderately differentiated	IIC	79
ovary	High-grade serous	3, poorly differentiated	IA	52
ovary	High-grade serous	3, poorly differentiated	IB	82
ovary	High-grade serous	3, poorly differentiated	IC	71
ovary	High-grade serous	3, poorly differentiated	IC	60
ovary	High-grade serous	3, poorly differentiated	IC	52
ovary	High-grade serous	3, poorly differentiated	IC	48
ovary	High-grade serous	3, poorly differentiated	IIB	78
ovary	High-grade serous	3, poorly differentiated	IIC	50
ovary	High-grade serous	3, poorly differentiated	IIC	54
ovary	High-grade serous	3, poorly differentiated	IIIB	83
ovary	Clear cell	2, moderately differentiated	IC	64
ovary	Clear cell	3, poorly differentiated	IC	82
ovary	Clear cell	3, poorly differentiated	IC	50
ovary	Endometrioid	2, moderately differentiated	IC	69
ovary	Endometrioid	3, poorly differentiated	IA	48
ovary	Endometrioid	4, undifferentiated	IA	74
ovary	Mucinous	2, moderately differentiated	IA	63
ovary	Low-grade serous	1, well differentiated	IIC	82
